# Supplementary material for: Redundancy circuits of the commissural pathways in human and rhesus macaque brains
Source: Hum Brain Mapp. 2021 Feb 9;42(7):2250–61. doi: 10.1002/hbm.25363 (PMC8046059; doi:10.1002/hbm.25363)
Supplement: Supplementary file 1 — Supplementary Figure 1 Protocol for mapping the anterior commissure for human and rhesus macaque subjects Supplementary Figure 2: Anterior commissure and corpus callosum visualization in the human brain Supplementary Figure 3: Anterior commissure and corpus callosum visualization in the rhesus macaque brain Supplementary Figure 4: Inverted human cadaveric dissection images Supplementary Figure 5: Inverted rhesus macaque cadaveric dissection images Supplementary Table 1: Regions the anterior commissure projects to in the human and rhesus macaque brain Supplementary Table 2: Abbreviations used in the dissection images [file HBM-42-2250-s003.docx]

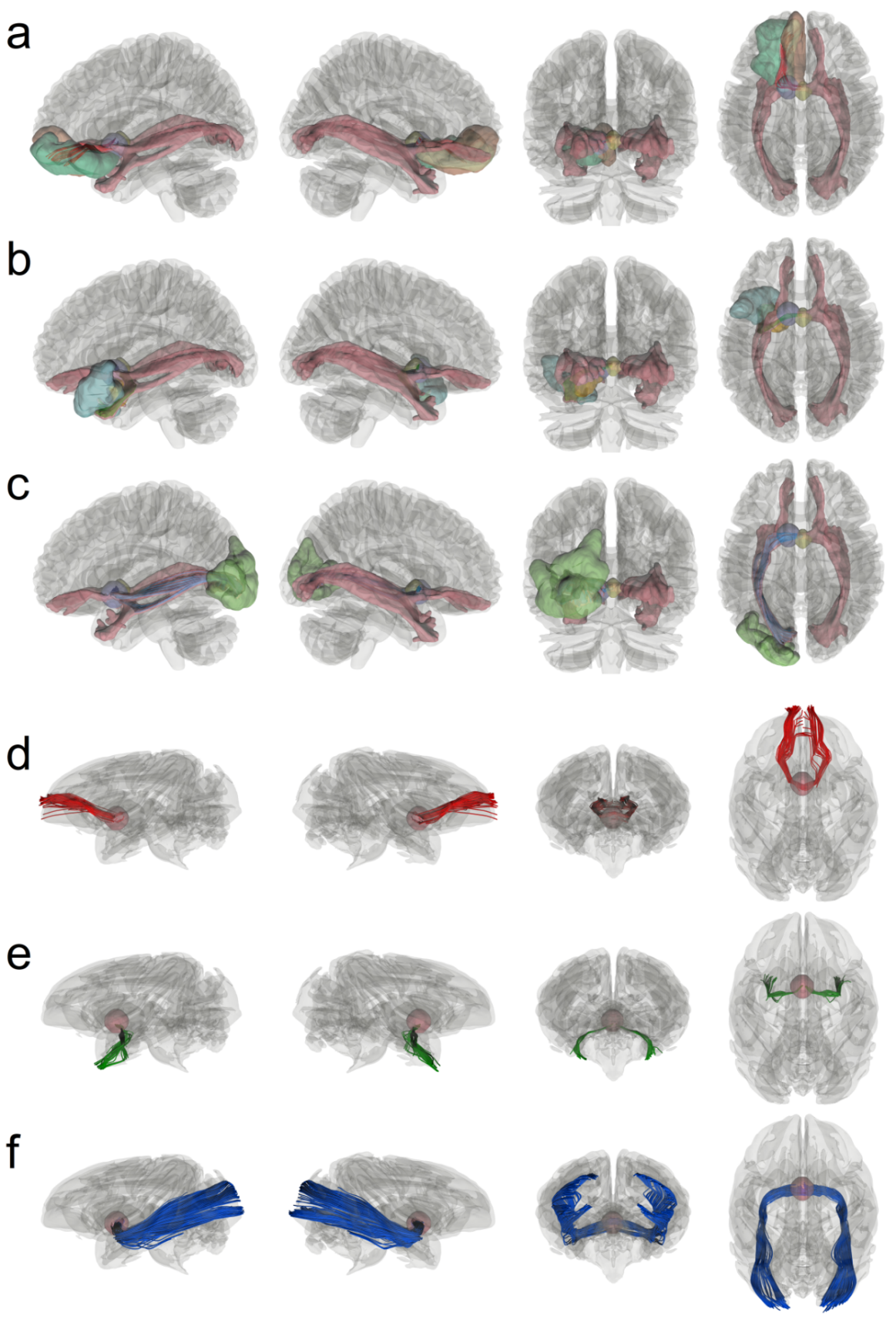


**Supplementary Figure 1:** Protocol for mapping the anterior commissure for human and rhesus macaque subjects


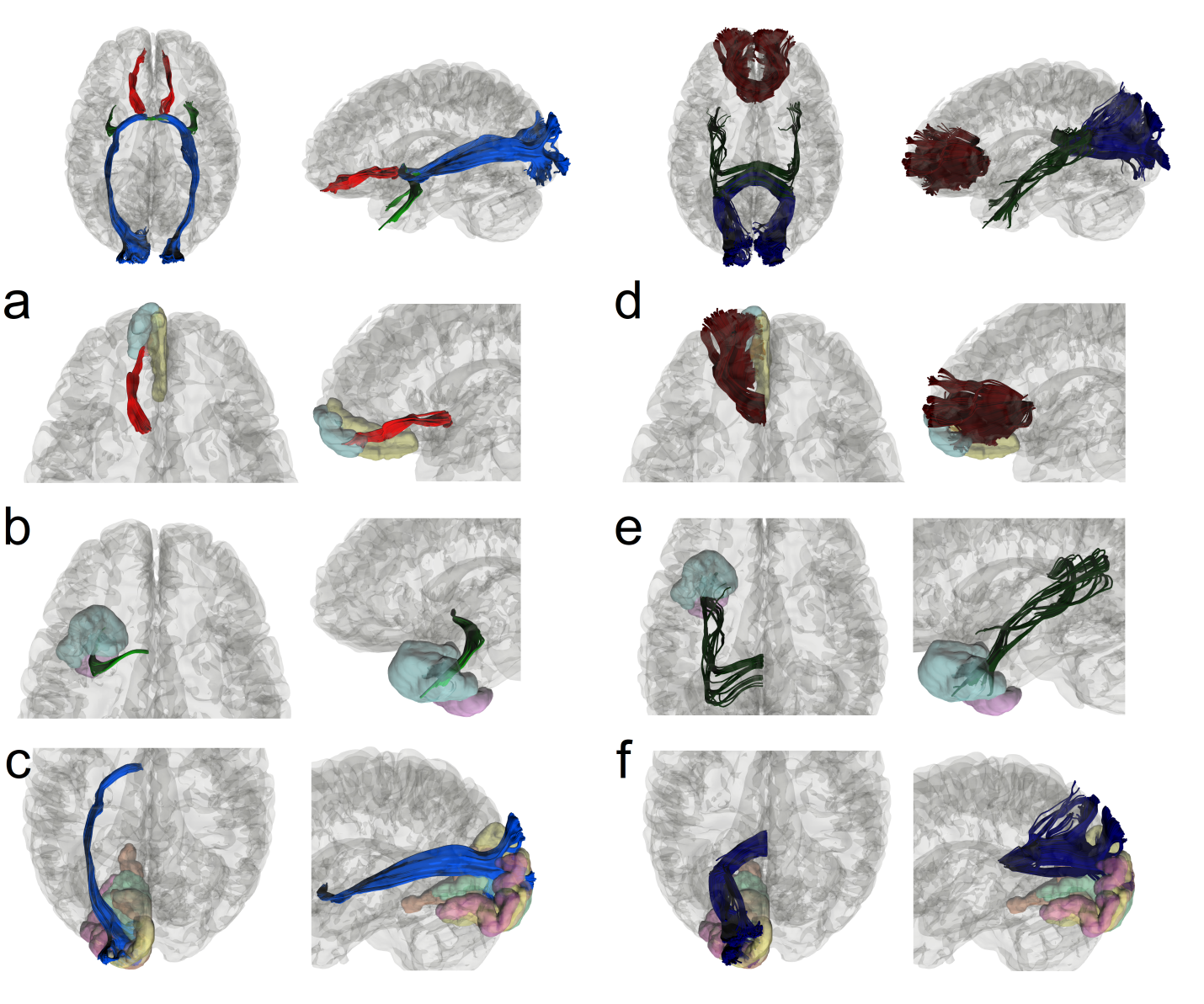
**Supplementary Figure 2:** Anterior commissure and corpus callosum visualization in the human brain


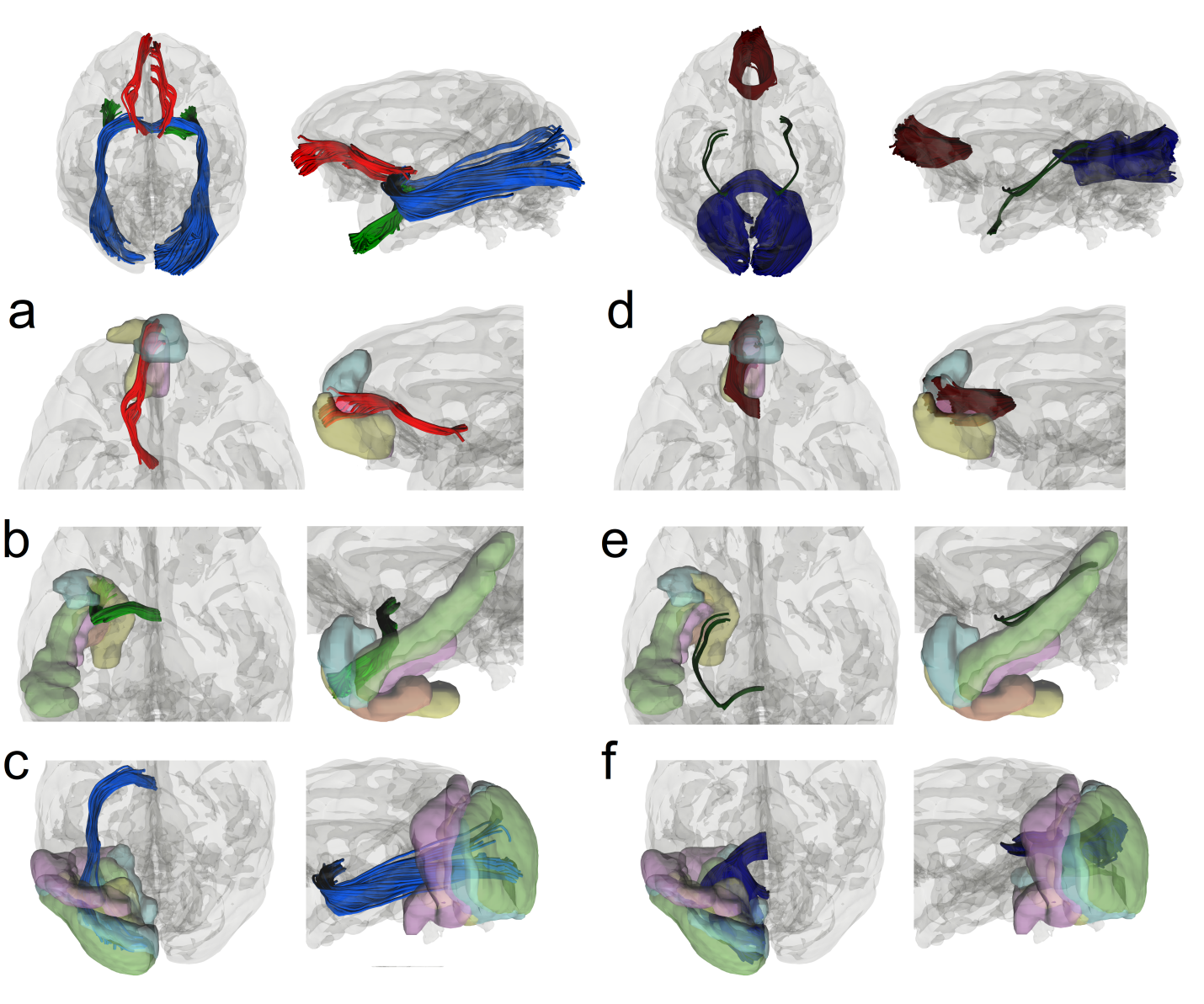
**Supplementary Figure 3:** Anterior commissure and corpus callosum visualization in the rhesus macaque brain


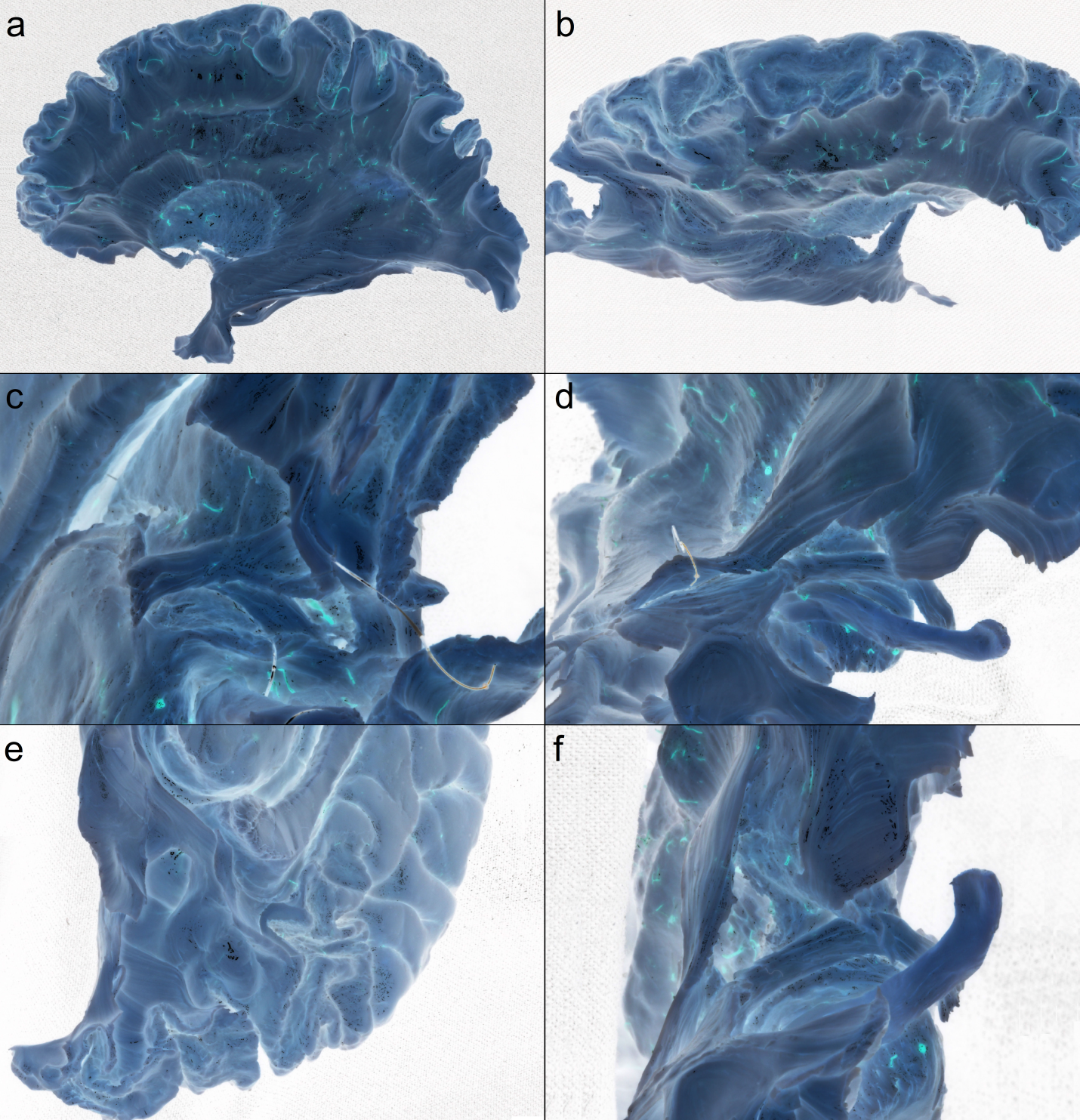
**Supplementary Figure 4:** Inverted human cadaveric dissection images


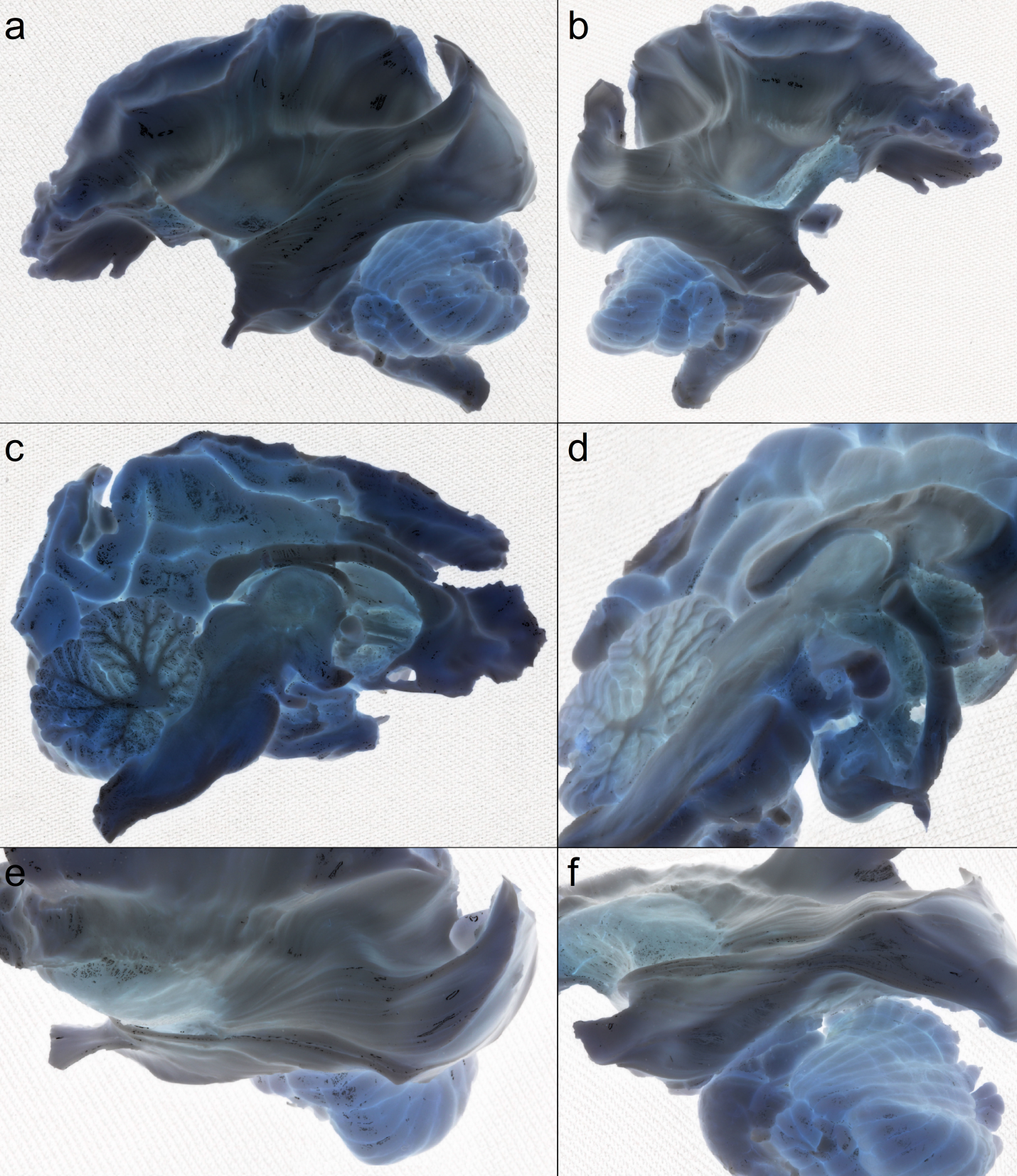


**Supplementary Figure 5:** Inverted rhesus macaque cadaveric dissection images

**Supplementary Table 1**: Regions the anterior commissure projects to in the human and rhesus macaque brain

|  | Occipital | Orbitofrontal | Temporal |
| --- | --- | --- | --- |
| Human | V1  V2  V3  V4 | 10v  10pp | TGd  TGv |
|  | | | |
| Rhesus macaque | V1  V2  V3  V4 | 10M  10V  14M | IPa  TLR (R36)  TE1  TPO  TPPro |

**V1:** primary visual cortex

**V2:** secondary visual cortex

**V3:** tertiary visual cortex

**V4:** quaternary visual cortex

**10v:** area 10 ventral

**10pp:** polar 10p

**TGd:** area TG dorsal

**TGv:** area TG ventral

**10M:** area 10 of cortex medial part

**10V:** area 10 of cortex ventral part

**14M:** area 14 of cortex medial part

**IPa:** intraparietal sulcus associated area in the superior temporal sulcus

**TLR(R36):** area TL rostral part (area 36R)

**TE1:** temporal area

**TPO:** temporal parietoccipital associated area in sts

**TPPro:** temporopolar prisocortex

**Supplementary Table 2:** Abbreviations used in the dissection images

| **Abbreviations** | **Brain Regions** |
| --- | --- |
| AC | Anterior commissure |
| AnsPed | Ansa Peduncularis |
| VA | Ventral anterior nucleus |
| Calc Fiss | Calcarine Fissure |
| CN | Caudate Nucleus |
| CC | Corpus callosum |
| CB | Cerebellum |
| CR | Corona Radiata |
| FB | orbitofrontal branch |
| F | Fornix |
| HP | Hippocampus |
| IFOF | Inferior Front-Occipital Fasciculus |
| Lent | Lentiform nucleus |
| M | Mammillary bodies |
| OB | Occipital branch |
| Opt | Optic nerve |
| POSulc | Parietooccipital Sulcus |
| P | Pons |
| PreCun | Precuneus |
| PT | Putamen |
| RectG | Rectus Gyrus |
| SLF | superior longitudinal fasciculus |
| TH | Thalamus |
| TB | Temporal branch |
| UN | Uncinate fasciculus |
| V1 | primary visual area |
| V2 | secondary visual area |
| V3 | tertiary visual area |
