# Supplementary figures and images for: Redundancy circuits of the commissural pathways in human and rhesus macaque brains
Source: Hum Brain Mapp. 2021 Feb 9;42(7):2250–61. doi: 10.1002/hbm.25363 (PMC8046059; doi:10.1002/hbm.25363)

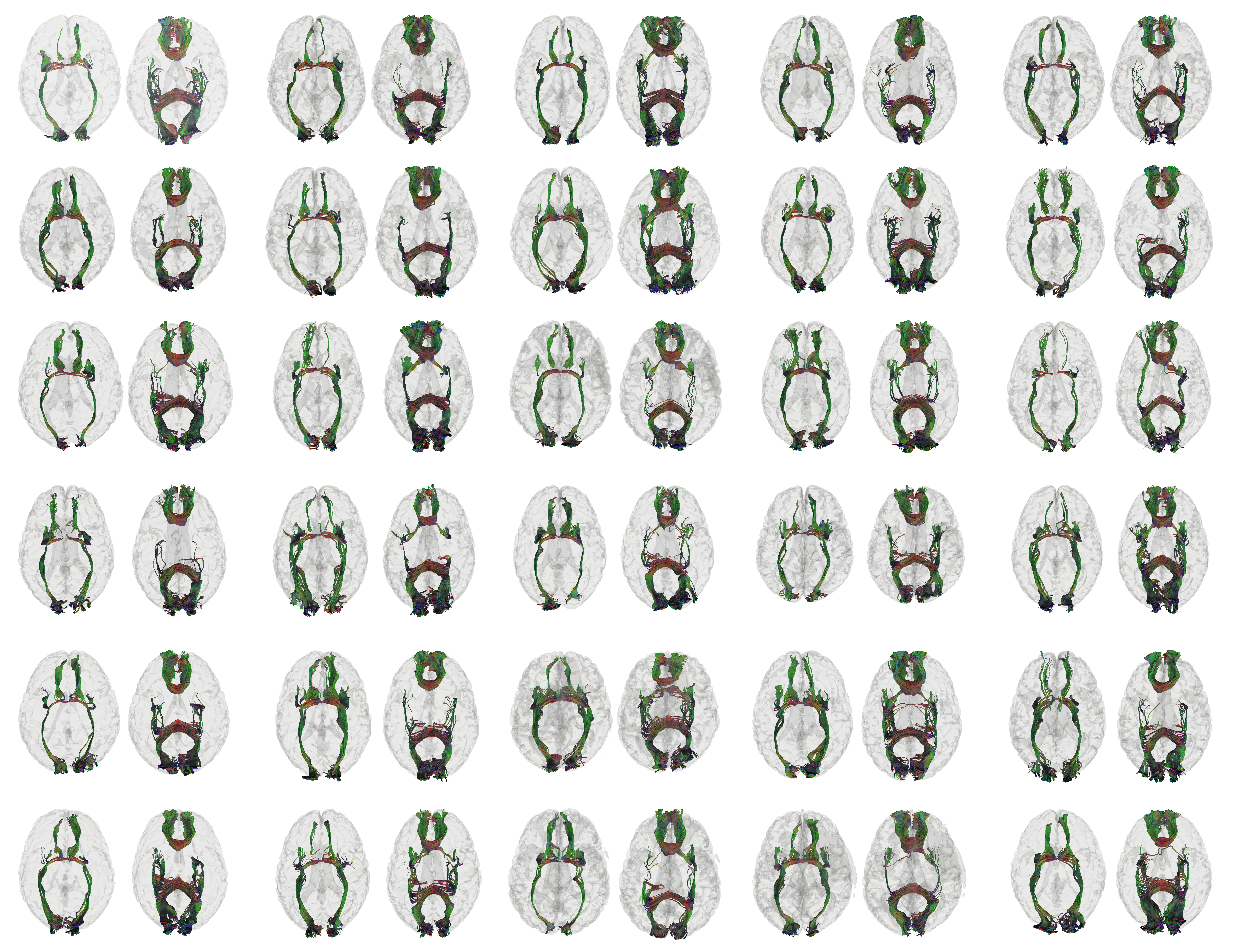

Supplement: Supplementary file 2 — Supplementary Figure 6 Rhesus macaque tractography results for each subject [file HBM-42-2250-s001.tif]

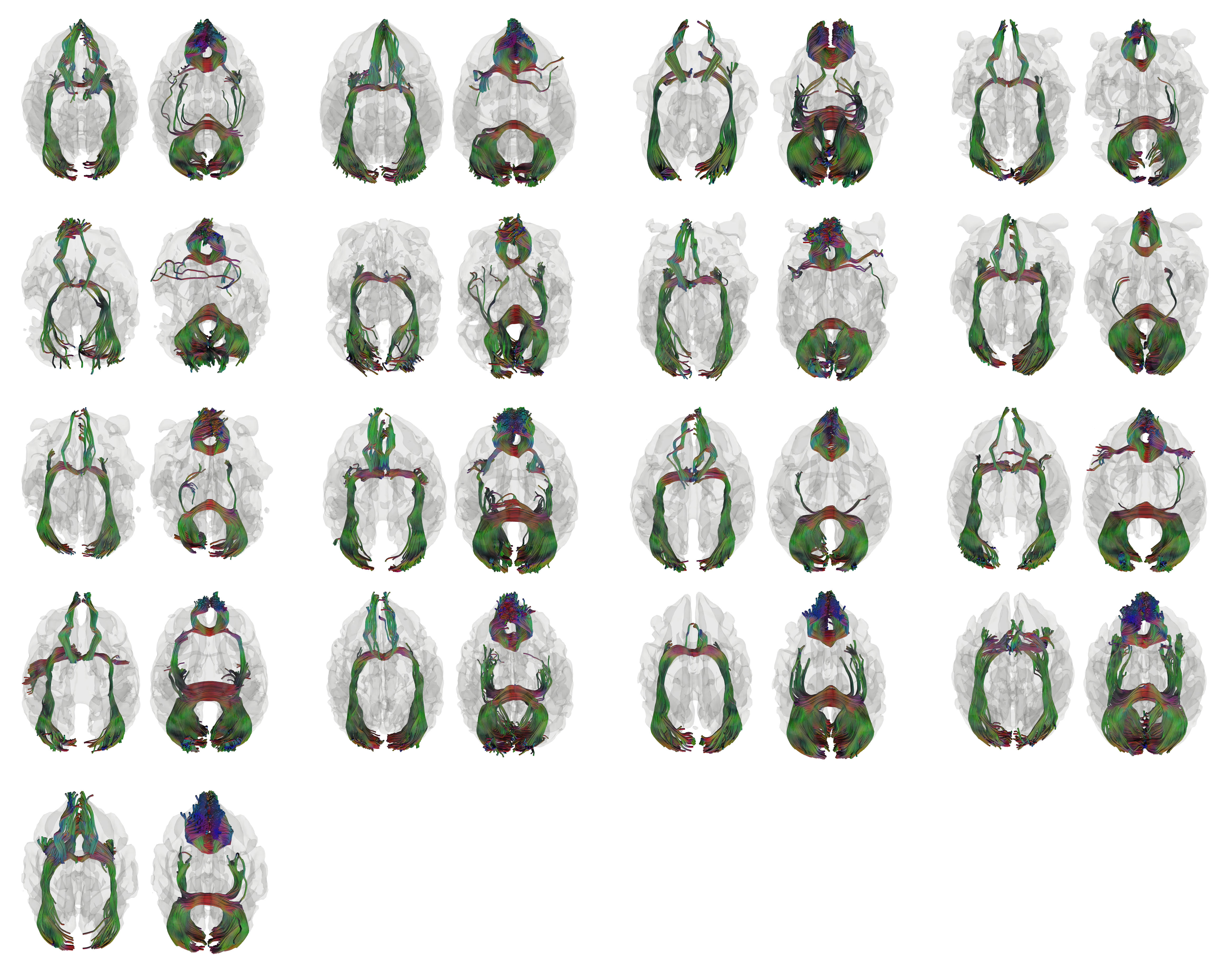

Supplement: Supplementary file 3 — Supplementary Figure 7 Rhesus macaque tractography results for each subject [file HBM-42-2250-s002.tif]
